# Cannabidiol polarizes human neutrophils toward a cancer-promoting phenotype

**DOI:** 10.3389/fimmu.2025.1543403

**Published:** 2025-07-25

**Authors:** Mona Khoury, Yuxiang Hong, Dayana Blokon-Kogan, Stela Gengrinovitch, Harel Eitam, Moran Avraham-Kelbert, Hadas Weinstein-Marom, Peng Xu, Idan Cohen, Gil Bar-Sela

**Affiliations:** ^1^ Cancer Center, Emek Medical Center, Afula, Israel; ^2^ Bruce Rappaport Faculty of Medicine, Technion-Israel Institute of Technology, Haifa, Israel; ^3^ Department of Chemical Engineering, Guangdong Technion-Israel Institute of Technology (GTIIT), Shantou, Guangdong, China; ^4^ Laboratory of Immunology, MIGAL-Galilee Research Institute, Kiryat Shmona, Israel; ^5^ Dept. of Biotechnology, Tel-Hai College, Kiryat Shmona, Israel; ^6^ Clalit Clinical Laboratories, Emek Medical Center, Afula, Israel

**Keywords:** primary neutrophils, cannabinol (CBD), neutrophil activation, cytokine secretion, NETosis

## Abstract

**Introduction:**

Cannabidiol (CBD) is widely used as a natural alternative supplementary treatment for side effects and symptom relief in many diseases. Although the benefits and risks of using CBDs are still largely unknown, consumption has grown constantly.

**Methods:**

Primary human neutrophils were isolated and exposed to CBD. Neutrophil functions such as oxidative burst, cytokine and chemokine production, bacterial killing, NET formation, and expression of cell surface markers were assessed. Conditioned media (CM) from cells treated with or without CBD were collected, and their impact on cancer cell proliferation, migration, and angiogenesis was examined. Furthermore, Neutrophil/T-cells co-culture was conducted to determine their effects on T-cell proliferation and activation.

**Results:**

We show that CBD induces human primary neutrophils to polarize into an N2-like cancer-promoting phenotype. CBD-exposed neutrophils exhibit reduced oxidative burst, reduce bacterial killing, and altered the production of cytokine and chemokine arrays like N2-polarized cells. CBD-treated cells also rapidly display a landscape of surface markers compatible with the described setup, known for N2-polarized cells, and promote cancer cell proliferation, migration, angiogenesis, and boost the expression of PD-L1 in cancer cells. Furthermore, CBD-stimulated neutrophils suppressed T-cell proliferation, suggesting that this signalling pathway may be involved in regulating T-cell antitumor immunity and immunotherapy.

**Discussion:**

Our study highlights a potential risk of CBD use in cancer patients and underscores the need for further investigation into its immunological effects and signalling mechanisms.

## Introduction

The endocannabinoid system (ECS) is a complex signaling network that regulates various physiological processes (1). This intricate system maintains balance and homeostasis from mood and pain sensations to appetite, sleep, and immune responses, and can be modulated by specific endogenous and exogenous receptor-interacting cannabinoids (2, 3). Cannabidiol (CBD) is one of the primary nonpsychoactive cannabinoids found in *Cannabis sativa* and is known to interact with endogenous cannabinoid receptors (CBRs). CBD can transduce signals via subtle interactions with two primary cannabinoid receptors, CBR1 and CBR2, as well as other components of the ECS (4, 5). These properties hold promise for CBD as a therapeutic agent in various applications, ranging from neurological and psychological diseases to inflammatory and autoimmune diseases, as well as oncological conditions (5–7). Indeed, backed up by multiple clinical trials, CBD consumption now has a diverse range of therapeutic benefits, encompassing antimicrobial and antiepileptic action, neuroprotection, anxiety reduction, antipsychotic effects, and pain relief, and may even help to combat cancer (5, 6, 8). In line with these findings, during the last decade, various pharmaceuticals derived from cannabis compounds have gained approval for different medical uses from the Food and Drug Administration (FDA) and the European Medicines Agency (EMA). For example, Epidiolex (FDA and EMA-approved), a purified CBD extract, treats seizures linked to Lennox–Gastaut syndrome and Dravet syndrome. In certain countries, an oral spray called nabiximols containing THC and CBD is approved for multiple sclerosis-related spasticity and pain. Similarly, the legalization and consumption of CBD have undergone significant changes worldwide, with CBD products now fully legalized for medicinal or recreational use in many countries, recognizing their potential therapeutic benefits. As a result of the changing regulatory landscape, the global market for CBD products has witnessed remarkable growth in recent years, expanding beyond niche markets to become widely accessible. According to Forbes’ article published on Jul 28, 2021, the global CBD market is predicted to reach 19.5 billion USD by 2025.

As such, during the last COVID-19 pandemic, research into the potential benefits of CBD consumption demonstrated that CBD inhibits SARS-CoV-2 replication by introducing host ER stress and innate immune responses. Notably, CBD showed a significant negative association with positive SARS-CoV-2 test results in a matched group of patients, highlighting its potential preventive effects on early-stage SARS-CoV-2 infection (9). Likewise, numerous studies have demonstrated the immunomodulatory properties of CBD, including its ability to suppress the production of proinflammatory cytokines or chemokines, inhibit immune cell activation, and induce immune cell apoptosis ex vivo or *in vivo* in animal models. Furthermore, CBD also potently induced regulatory T-cells (T-regs) and immunosuppressive myeloid-derived suppressor cells (MDSCs) and was shown to decrease or inhibit leukocyte recruitment, migration, and chemotaxis [reviewed previously (4)]. Despite substantial persuasive findings confirming the immunomodulatory properties of CBD, most of these studies were primarily performed in animal models, leaving extensive gaps in our understanding of how CBD affects innate and adaptive immune cells or how it may modulate the human immune system, particularly during infections or inflammatory and autoimmune diseases. For instance, although the immunomodulating effects of CBD are mediated in a concentration- and cell type-dependent manner, the relationship between CBD dosing and immune modulation in a given immune cell type has not been examined. Likewise, it is currently unclear which component (s) of the ECS are responsible for CBD’s broad immunosuppressive effect, as no specific receptor(s) have yet been directly linked to a specific immune response. Moreover, the intracellular and molecular signaling pathways activated following transient or chronic CBD exposure are generally undefined and, in particular, remain unexplored in human primary immune cells (5, 10).

Neutrophils are the most abundant circulating leukocytes in humans; their ability to rapidly respond and eliminate invaders helps prevent the spread of infections, mostly by phagocytosing invading microorganisms, such as *S. aureus* (11). Moreover, as extremely short-lived cells (12), they have even essential functions after commitment to apoptosis, orchestrating macrophage functionality during the resolution phase of inflammation or by producing pro-resolving mediators themselves (13). Since most clinical and preclinical data on the effects of CBD on the human immune system are relatively scarce and outdated, they do not align with our current modern understanding of the immune system (14–16). Here, we utilized negative selection cell sorting to isolate highly pure primary human neutrophils (17) as opposed to polymorphonuclear cells (PMNs) from whole blood, enabling us to thoroughly examine the effect of CBD on human neutrophils.

## Materials and methods

### Subjects and ethical approval

All study participants (n=40) were ≥18 years old and healthy, with no abnormal blood counts or specific medical diagnoses. Every donor was informed before signing a consent form for blood sampling under the approval of the institutional Helsinki committee (0093-19-EMC). Peripheral blood samples were collected in ethylenediaminetetraacetic acid (EDTA) blood collection tubes immediately before neutrophil isolation and ex vivo experiments.

### Primary human neutrophil isolation and tissue culture maintenance

As previously described and tested by Salti et al., (17), human neutrophils were isolated via negative selection cell sorting using an EasySepTM Direct Human Neutrophil Isolation Kit (STEMCELL Technologies). According to the manufacturing procedure, highly purified primary human neutrophils were directly isolated from 3–20 ml whole-blood samples. The cells were maintained in RPMI 1640 medium supplemented with 2% fetal bovine serum (FBS), 1% penicillin/streptomycin, and 1% L-glutamine (all purchased from Biological Industries, Israel) and maintained at 37°C with 5% CO2. The cancer cell lines HeLa, HepG2, and A549 were cultured in Dulbecco’s modified Eagle medium (DMEM) (Biological Industries, Israel) supplemented with 5% FBS, 1% penicillin/streptomycin, and 1% L-glutamine at 37°C and 5% CO2.

### Apoptosis quantification

In all experiments, an equal volume of DMSO was added as a CBD negative control to account for any potential solvent effects and indicated as Control. To quantify and assess cell viability and apoptosis rates, we preincubated 1×106 neutrophils with different concentrations of CBD (5 μM to 120 μM) for 60 min. Inhibition of CBR-1, CBR-2, or GPR55 was performed by pretreating the cells with a CBR-1 antagonist (AM251, 5 μM), a CBR-2 antagonist (SR144528), or a GPR55 antagonist (CID-16020046) for 30 min before exposure to CBD. The cells were washed twice and stained with Annexin V-FITC and PI-PE MEBCYTO^®^ Apoptosis Kit (Annexin V-FITC Kit, MBL, Nagoya, Japan). Fluorescence intensities were measured using fluorescein isothiocyanate (FITC) and polymerase chain reaction (PE) filters in fluorescence-activated cell sorting (FACS) (NAVIOS Analyzer, Beckman Coulter, Inc.), and the data are presented as the means ± SEMs of 3–5 donors.

### Monitoring reactive oxygen species

Oxidative bursts and Intracellular ROS production were assessed using a dihydrorhodamine 123 (DHR 123) (Sigma) assay. 5×105 highly purified primary neutrophils were supplemented with DHR at a final concentration of 100 μM and incubated for 10 min at 37°C with gentle shaking. Oxidative bursts were induced with either PMA (100 nM) or lipopolysaccharide (LPS) (25 μg/ml) for 30 min with or without 12.5 μM CBD. The cells were centrifuged for 5 min at 2000 rpm at 4°C, followed by two washes with ice-cold PBS. Fluorescence intensities were measured via FACS using a FITC filter. The data were processed with FlowJo™ v10.8 Software (B.D. Life Sciences) and are presented as the mean -/+ SD from 3–5 donors.

Extracellular ROS was assessed using a hydrogen peroxidase assay kit (Cayman). A total of 1.5 ×105 highly purified primary neutrophils were seeded in 96 wells for 4 h to allow the cells to adhere, followed by incubation with or without GPR55 antagonist (CID-16020046) for 30 min. Cells were then centrifuged for 5 min at 2000 rpm at room temperature (RT). Supernatants were discarded, and the cells were incubated with fresh serum free medium, with or without CBD at 12.5 µM for 15 serum-free medium, with or without CBD at 12.5 µM for 15 min, along with the cell assay buffer according to the manufacturer’s instructions. Subsequently, the cells were stimulated with or without LPS (25 µg/ml) or PMA (100 nM). Immediately after enzyme activation buffer (HRP substrate-based reaction with Amplex Red bound to H2O2) was added and incubated for 5 min on a shaker. The absorbance was then measured at 570 nm using a plate reader (Infinite 200 PRO). The data are presented as the mean ± SD from 3–5 donors.

### Real-time qPCR gene expression

To evaluate the effect of CBD on gene expression, neutrophils were preincubated with or without 12.5 μM CBD for 60 min and then stimulated with 100 ng/ml LPS (Sigma). Differential changes in the gene expression of anti- or proinflammatory factors were assessed after 120 min. Total RNA was extracted from 2X107 neutrophils using the Quick-RNA Microprep Kit (Zymo Research). cDNA was generated from 1 μg of total RNA with a reverse transcription kit for high-capacity cDNA synthesis (Thermo Fisher Scientific, Inc.). Specific gene expression was evaluated by real-time quantitative polymerase chain reaction (qPCR) performed with a CFX96 Touch Real-Time PCR Detection System (Bio-Rad Laboratories, Inc.) using qPCRBIO SyGreen Blue Mix (PCR Biosystems Ltd., Wayne, Pennsylvania, USA). All qPCR data were normalized to the expression of the housekeeping gene human β-actin and are presented as the means/+ SDs of normalized expression taken from 4 different donors. The gene-specific primers used are listed in the Supplementary STAR method file.

### Protein extraction

Primary neutrophil 1×107 cells were plated in 10 cm culture dishes, treated with or without 12.5 μM CBD for 30 min, and stimulated with LPS 5µg/ml for 120 min. The cells were then collected by 5 min centrifugation at 2000 RPM at 4°C, followed by two washes with ice-cold 1XPBS. The cell plates were resuspended in 120 μL of RIPA buffer (50 mM Tris-HCl pH=7.5, 1% SDS, and 10% (v/v) containing protease inhibitors (p8340; Sigma–Aldrich, MO), 1% Triton X-100, and 150 mM NaCl) and incubated for 30 min on ice. We further disrupted the pellets with 3–5 gentle sonication pulses (Q125, Qsonica) to eliminate insoluble aggregates. Cell debris was removed by 10 min of centrifugation at maximum speed (12,000 RPM), and the total concentration of cell soluble proteins was measured with a Bradford assay (Bio-Rad).

### Human MAPK phosphorylation antibody array and phospho-gsk3 monitoring

For pathway analysis and quantitative measurement of the relative levels of human phosphorylated MAPK kinase activity, whole-cell soluble protein extracts from 1×107 highly purified primary human neutrophils were preincubated with or without 12.5 μM CBD (30 min) and then stimulated with 5 µg/ml LPS (Sigma) for 120 min. According to the manufacturer’s instructions, equal amounts of total soluble proteins were allowed to react with the Human MAPK Phosphorylation Antibody Membrane Array (ab211061, Abcam). Membrane visualization and signal quantification were performed using the G-BOX F3 Gel Doc-System (Syngene). To validate the GSK3a phosphorylation status, we used phospho-GSK3α cell-signaling and general anti-GSK (cell-signaling) antibodies, followed by incubation with a secondary antibody conjugated to PE (Jackson). Signals were measured via FACS (NAVIOS Analyzer, Beckman Coulter, Inc.) and are presented as the means -/+ SDs; data were obtained from 3 donors.

### Bacterial killing assay

*Escherichia coli* (*E. coli*) and *Staphylococcus aureus* (*S. aureus*) were isolated from single-celled ATCC bacterial strains (kindly gifted by the Emek Hospital Microbiology Unit) from isolated overnight Luria–Bertani (LB) broth agar plates. Both bacterial cultures were allowed to grow overnight in LB, diluted 1/20 in fresh LB media, and grown to achieve logarithmic phase growth of 0.1 to 0.3 optical density (OD) at 600 nm for approximately 120 min. Since CBD is known to have potent broad-range antimicrobial activity against various gram-negative and gram-positive bacteria (18), we avoided the presence of CBD in any media containing bacteria. As previously described (17), freshly isolated neutrophils were preincubated with 12.5 μM CBD (60 min), centrifuged at 2000 rpm for 5 min, and washed twice in 1X PBS. For neutrophil bacterial killing, 1×106 bacteria and 1×106 neutrophils were resuspended in fresh RPMI 1640 medium (without antibiotics) at a 1:1 ratio and incubated for 1 hour. The samples were finalized by serially diluting the samples in double-distilled sterile water and spreading them on agar plates. Colony-forming units (CFU) were manually counted in triplicate for each sample, and bacterial killing was calculated compared to the number of CFUs resulting from a control sample of bacterial culture incubated without neutrophils. The data are presented as the means -/+ SDs of 3 donors.

### Extracellular NET cell-free DNA quantification

Primary human neutrophils (2×105 cells) were seeded in 96-well plates (SPL Life Science, Korea). The cells were treated with or without 12.5 μM CBD for 30 min, followed by stimulation with or without 200 nM PMA or 5 μg/ml LPS for 240 min. The plates were centrifuged at 2000 rpm for 5 min to clear the supernatants from the cell debris. A total of 100 mL of cell supernatant was collected, and cfDNA was measured by staining with SYTOX™ Green Nucleic Acid Stain (Invitrogen), and the fluorescence intensity was measured at Excitation 485/Emission 523 with a plate reader (Infinite 200 PRO). All the experiments were performed in duplicate, and the data are presented as the means (in -/+) and SDs. of relative fluorescence units (RFUs) obtained from 3–5 donors.

### Confocal microscopy

Primary neutrophils (4×105) were seeded in each well on 8-chamber slides (Lab-Tek) and allowed to adhere for 60 min. The cells were treated with or without 12.5 μM CBD for 30 min and then stimulated with either 100 nM PMA or 5 μg/ml LPS for 240 min. The samples were fixed with 4% paraformaldehyde for 10 min, permeabilized with Triton X-100 (0.1% v/v) for 5 min, and stained with SYTOX™ Green Nucleic Acid Stain (Invitrogen). Imaging was performed using a wide-20 field confocal microscope (Zeiss LSM 880).

### Giemsa staining

Primary neutrophils (1×106) were seeded in 6 wells with or without CBD 12.5µM for 1.5 h or 3 h. Following incubation, cells were cytospin onto slides by the Aerospray Hematology Pro Slide Stainer (ELITechGroup) for 5 minutes. Smeared slides were fixed in methanol for 5 min and dried. Then, the slides were stained with Giemsa stain according to the manufacturer’s instructions (Sigma-Aldrich). Snapshots were taken using a light microscope (Olympus BX51) with a 100X objective from 3 individual donors.

### Flow cytometry

As previously described, highly pure isolated primary human neutrophils were incubated with the indicated stimulators. 1X106 cells were washed twice with Flow Cytometry Staining Buffer (Thermo Scientific), and the Fc receptor was blocked for 10 min using Human Truestain FcX™ (blocking step) (BioLegend) to reduce nonspecific background antibody signals. For neutrophil surface receptor expression after CBD (12.5 μM for 150 min) exposure, we used the following antibodies: APC-conjugated anti-human CD62L (BioLegend), PE-conjugated anti-human CD182 (CXCR2) (BioLegend), KIRAVIA Blue 520™ anti-human CD95 (Fas) (BioLegend), APC-conjugated anti-human CD54 (BioLegend), FITC-conjugated anti-human CD11b (Beckman Coulter), and APC-conjugated anti-human CD66b (Invitrogen). The cells were washed and analyzed separately for each antibody. Phosphoglycerogen synthase kinase alpha (GSK3α), phospho-GSK-3α (Ser21) (36E9), rabbit mAb #9316, phospho-GSK-3α/β (Ser21/9), antibody #9331 and GSK-3β (D5C5Z) XP rabbit mAb #12456 were all purchased from Cell Signaling Co. and used to assess GSK phosphorylation and overall protein levels. The cells were then washed with FACS buffer, stained with anti-rabbit IgG-PE (Jackson), and resuspended in 1X PBS before analysis using a NAVIOS flow cytometer (Beckman Coulter, Inc.). The mean fluorescence intensity (MFI) was recorded for at least 100,000 cells and is presented as the average −/+ standard deviation (SD) of 3 independent donors.

### Multiplex enzyme-linked immunosorbent assay

For the detection and quantification of specific secreted cytokines, 5×105 primary neutrophils were seeded in 24-well dishes and preincubated with the following inhibitors or antagonists: 10 μM AM251, 10 μM SR144528, or 10 μM BCTC with or without 12.5 μM CBD for 30 minutes. To induce cytokine secretion, cells were stimulated with 100 ng/ml LPS (Sigma) or incubated with heat-killed (15 min at 70°C) *S. aureus*, K. pneumoniae, or Candida albicans at a 1:1 ratio or with 1× PBS (as a control) for an additional 120 minutes. The cytokines secreted into the cell supernatants were assessed with the Multiplex Human Luminex Discovery Assay (5 PLEX) (A B-05-LXSAHM) for the following cytokines: chemokine (C-X-C motif) ligand 2 (CXCL2/GRO beta/MIP-2/CINC-3), interferon beta (IFN-β), interleukin-1 (IL-1) IL-1β/IL-1F2, IL-8/CXCL8, and TNF-α; all of which were performed by American Medical Laboratories (AML Israel). The data are presented as the means −/+ SDs obtained from 5 independent donors.

### *In-vitro* cancer assays

For all cancer-based *in vitro* assays, we first collected neutrophil-conditioned medium from 1×107 cells incubated with or without CBD for 2 hours. The cells were then thoroughly washed three times in 1X PBS (to remove traces of CBD). Then resuspended in 4×106 cells/ml in fresh neutrophil growth medium (RPMI 1640, 2% FBS, 1% penicillin/streptomycin, and 1% L-glutamine) and maintained at 37°C with 5% CO2 for 24 to 48 hours. The conditional medium (CM) of -/+ CBD-stimulated neutrophils was collected, and cells or debris were removed by centrifugation at 12,000 rpm for 5 min following filtration with a 0.5 μm filter. All cancer cell lines, HeLa (cervical cancer), HepG2 (hepatocellular carcinoma), and A549 (human non-small cell lung cancer), were first grown in standard DMEM containing 10% FBS, 1% penicillin/streptomycin, and 1% L-glutamine (purchased from Biological Industries, Israel) at 37°C with 5% CO2. For the proliferation assay, 2 ×104 to 5×104 cells per well were resuspended in -/+ CBD-stimulated neutrophil conditioned medium (96-well plates). Ratios of cell growth were quantified using an XTT-based Colorimetric Cell Proliferation Kit (Sartorius). Not that due to CBD’s high lipophilicity and strong protein-binding affinity, minimal residual CBD may remain in the cell medium.

For the cancer cell migration assays, all cancer cell lines were first starved by resuspending them in a serum-free growth medium for 24 hours. Next, 1-4×104 cells were seeded into the upper chamber of an 8‐μm pore Transwell (SPL Life Sciences). We used 500 μL of neutrophil CM-/+ CBD for all migration experimental groups as a chemoattractant (lower chambers). As a control, 500 μL of serum-free medium was used. The cells were cultured in a humidified incubator at 37°C with 5% CO2 overnight. Transwell inserts were washed twice with PBS. The cells inside the transwell inserts were gently removed using moistened cotton swabs, and the cells on the lower surface of the membrane were then stained with crystal violet (Sigma) for 20 min, washed twice with PBS, and then air-dried before migrating cells were visualized by light microscopy.

Human umbilical vein endothelial cells (HUVECs) were kindly gifted from the Levenberg laboratory (Faculty of Biomedical Engineering, Technion). For the endothelial tube formation assay (angiogenesis assay), HUVECs (1.5×104 cells) were seeded on Matrigel (50 µl/well) in a 96-well plate at 37 °C with 5% CO2 and suspended in CM or CM-CBD for 6 h according to the instructions of the Angiogenesis Assay Kit (Abcam, ab204726). Five field snapshots per well (center of the well and four cardinal points) were taken using an inverted light microscope (Nikon) with a 10X objective in phase contrast mode without fixation. Snapshots were taken from 3 individual donor CMs and quantified using ImageJ for statistical analyses.

Neutrophil *in-vitro* cancer cell killing was performed as described in Bingwei et al., 2018, (19). Briefly, cells from each cancer cell line (e.g., HeLa, HepG2, and A549) were seeded in 96-well plates and allowed to adhere. CBD-treated or untreated washed neutrophils were cocultured with cancer cells at a 20:1 neutrophil-to-tumor cell ratio for 24 hours. Following incubation, the neutrophil anticancer cytotoxic activity was quantified using XTT assays, and neutrophil killing capacities were calculated relative to those of a parallel control cancer cell culture without neutrophils. To monitor changes in programmed death-ligand 1 (PD-L1) levels, cancer cells were cultured in CBD-treated/untreated neutrophil culture medium for 24 or 48 hours. The surface expression of PD-L1 was assessed via flow cytometry with a specific antibody (as described above). The results are presented as the means of fluorescence intensities (MFI) -/+ SDs taken from 3 different donors.

### PBMC culture

Human peripheral blood mononuclear cells (PBMCs) were obtained from ‘Cell Generation’ (BioPark, Jerusalem, IL) and cultured in RPMI 1640 supplemented with 10% fetal bovine serum (FBS), 4 mM GlutaMAX, 20 mM HEPES, 0.1 mM 2-mercaptoethanol, and 50 ng/ml recombinant human IL-2 (PeproTech, Cranbury, New Jersey). For activation, 5×105 PBMCs/ml were stimulated with 50 ng/ml anti-CD3 (BioLegend, San Diego, CA) and rhIL-2 and incubated for 48 hours. Once the cluster formed, the cells were washed twice with PBS and collected by centrifugation.

### T-cell activation and proliferation inhibition by T-cell–neutrophil coculture

PBMCs were stained with a CFSE Cell Division Tracker Kit (BioLegend) to assess proliferation according to the manufacturer’s protocol. Briefly, PBMCs were resuspended in 4 ml of PBS and stained with 2 μL of 5 mM CFSE per 20 million cells to yield a final CFSE concentration of 2.5 μM. PBMCs were incubated at room temperature for 20 min, after which a growth medium was added to neutralize the dye. The cells were centrifuged at 1250 RPM for 10 min, resuspended in growth medium at 1 million per ml, incubated for 10 min, and stored at 37°C until plated with the neutrophils. CFSE-stained PBMCs were cocultured with neutrophils at a 5:1 neutrophil: T-cell ratio in 12-well plates in complete T-cell medium with or without stimulation (50 ng/ml anti-CD3 antibody and 50 ng/ml anti-CD28 antibody; BioLegend, San Diego, CA). Proliferation analysis was performed after one and two days of coculture using a NovoCyte Quanteon Flow Cytometer (Agilent Technologies, Santa Clara, CA), and the data were analyzed with NovoExpress Software (Agilent). Three healthy donors present. The data are presented as the means +/- SDs. To assess T-cell activation markers, we used anti-human CD69-VioBlue (clone: REA824, Miltenyi), anti-human PD-1 (CD279-PE (clone: REA1165, Miltenyi), anti-human CD3-FITC (clone: UCHT1, Beckman Coulter), and anti-human CD137-APC (clone: REA765, Miltenyi). All the antibodies were stained at 2-8°C for 10 min; the cells were washed twice and analyzed in parallel with the CFSE-proliferation assay using FACS.

### Statistical analysis

GraphPad Prism 8.3.0 (GraphPad Software, Inc., La Jolla, CA) was used for the statistical analysis. Differences between groups (e.g., LPS vs. LPS + CBD) were tested utilizing a paired t-test at a p < 0.05 significance level. All data are presented as the means ± SDs. Statistical differences are indicated by asterisks ****p < 0.0001; ***p < 0.001; **p < 0.01; *p < 0.05.

## Results

Since CBD is known to induce apoptosis in murine monocytes, thymocytes, and primary human monocytic and leukemia cells (20–22) and has recently been reported to alter the functionality of PMNs, mainly by decreasing their viability (23), as a first step in examining the effect of CBD on human primary neutrophils, we tested whether CBD may cause neutrophil apoptosis. Therefore, we tested a range of CBD concentrations (5 μM to 120 µM) and assessed neutrophil viability and apoptosis levels using Annexin-V and propidium iodide (PI) staining to distinguish between different cell death pathways. In agreement with previous reports (23), we observed a significant decrease in neutrophil viability and a parallel increase in apoptosis after 1h of incubation with 15 µM CBD (or higher), reaching 50% viability at 120 µM CBD (**Supplementary Figure 1**). CBD-induced apoptosis was found to be independent of CBR1, CBR2, and G protein-coupled receptor 55 (GPR55) signaling, as neither the CBR1 antagonist (AM251), the CBR2 inverse agonist (SR144528), nor the GPR55 (CID-16020046) inverse agonist had any effect on cell apoptosis (**Supplementary Figure 1**). Therefore, 12.5 μM was selected as the initial CBD concentration that did not impact neutrophil viability, contrasting with earlier findings that reported morphological changes in PMNs at a concentration of 12.5 μM CBD. (23). We did not observe a significant alteration in the neutrophils’ overall morphology; cells appeared unchanged, with no apparent nuclear modifications besides a slightly stronger cytoplasmic Giemsa stain (**Supplementary Figure 2**).

**Figure 1:**
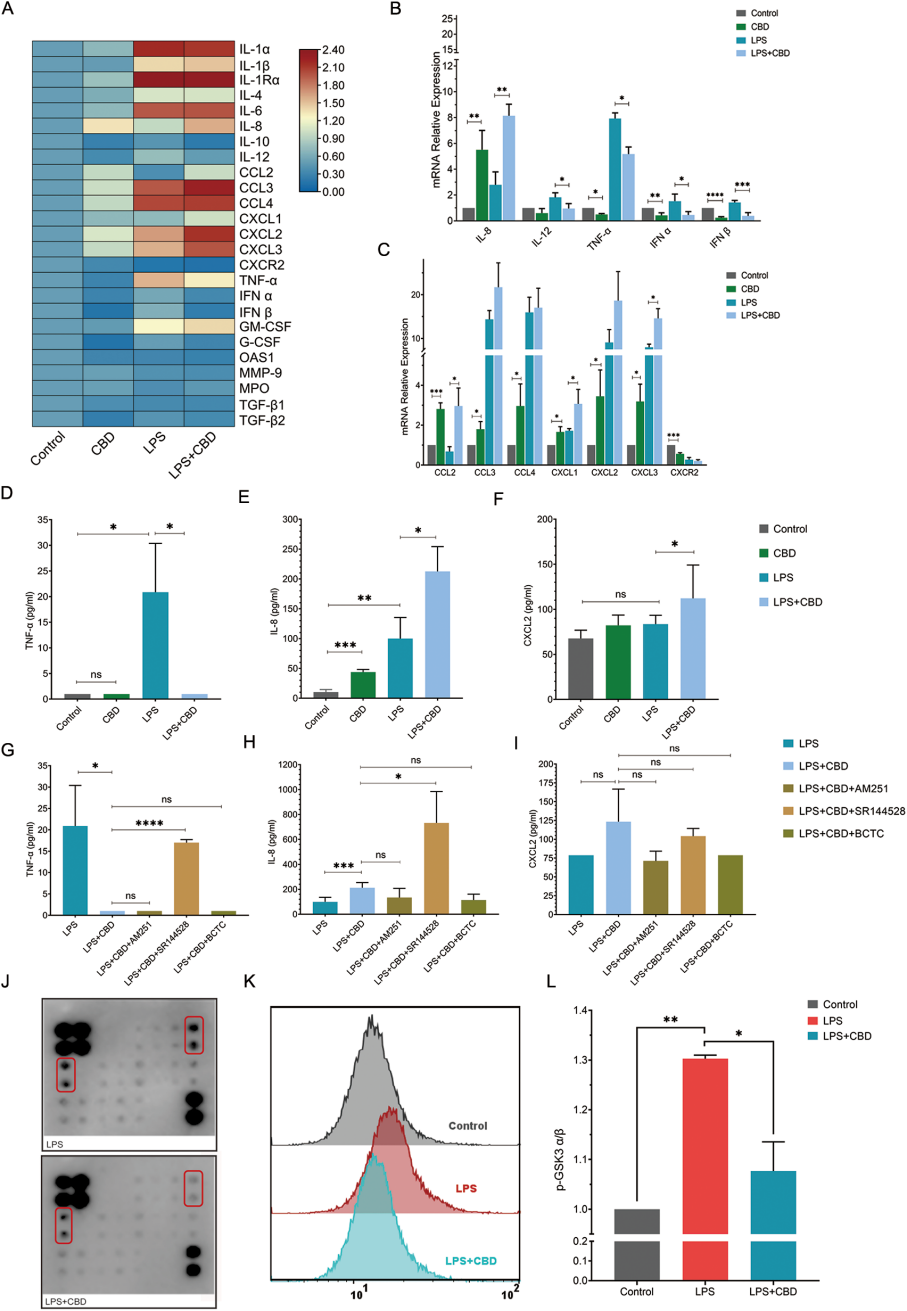
CBD and CBR2 signaling alter cytokine and chemokine production and secretion in human neutrophils. **(A)** Heatmap depicting changes in a panel of human neutrophil pro- and anti-inflammatory chemokines and cytokine expression levels in response to CBD exposure. The data are expressed as the mean (log_2_) normalized expression levels (means +/-SD of n =5). Validation and quantification of key pro- and anti-inflammatory **(B)** cytokines and **(C)** chemokines using qPCR. The expression levels of selected neutrophil-expressed genes showing changes in percent expression (normalized to control) for genes from resting (untreated) or activated (100 ng/ml LPS) neutrophils compared to cells preincubated with or without CBD (12.5 µM). ELISAs of total secreted cytokines for **(D)** TNF-α, **(E)** IL-8 and **(F)** CXCL2 were performed from the same e*x vivo* experiments as in **(B)** Cell supernatant ELISA was used to measure total secreted cytokine levels of **(G)** TNF-α, **(H)** IL-8 and **(I)** CXCL2 in resting or activated neutrophils with or without CBD (12.5 mM) in the presence of specific CBR1/2 or TRPV1/TRPM8 channel blockers (CBR1-AM251, CBR2- SR144528, BCTC- TRPV). **(J)** Array images comparison (one-sample analysis) of the human MAPK phosphorylation antibody array with total soluble protein extracts from LPS-activated (top) or neutrophils preincubated with CBD (LPS+CBD) (bottom). **(K)** Representative FACS histogram or **(L)** intracellular staining and FACS quantification (MFI -/+ SD, n=3) of phospho-GSK-3α (Ser21). As a control, we used intracellular staining with a total nonphosphorylated GSK antibody (see **Supplementary Figure 4**), which was performed on all the samples (n=3). * Significant p < 0.05; ** More significant p < 0.01; *** Highly significant p < 0.001; **** Very highly significant (less common) p < 0.0001; ns, Not significant p ≥ 0.05.

In line with the ability of CBD to suppress or reduce proinflammatory cytokine production in a dose-dependent manner through the activation of human mononuclear cells (0.03–64 μM CBD) (7, 22), we tested the cytokine production of primary human neutrophils in the presence of CBD. The cells were preincubated with or without CBD, and the neutrophils were activated with lipopolysaccharide (LPS) for two hours. The expression of a broad array of pro- or anti-inflammatory cytokines was assessed and compared with that of non-stimulated control cells (e.g., cells treated with or without LPS). Surprisingly, we did not observe significant changes in the transcript levels of key proinflammatory cytokines, such as IL-1α, IL-1β, or IL-6; granulocyte colony-stimulating factor (G-CSF); or the anti-inflammatory cytokines IL-10, TGF-β, and IL−4 (**Supplementary Figure 3**). Nonetheless, exposure to CBD significantly reduced INF-α, INF-β, IL-12, and tumor necrosis factor-alpha (TNF-α) expression levels (**Figures 1A, B**).

On the other hand, CBD alone or when combined with LPS, strongly induces the expression of IL-8 or chemokines such as CCL2 (monocyte chemoattractant protein-1 or MCP-1), CCL3 (MIP1α), CCL4 (MIP1β), and (C-X-C motif) ligand 1 (CXCL1); CXCL2 [macrophage inflammatory protein 2-alpha (MIP2-alpha)]; and CXCL3 [also known as GRO3 oncogene (GRO3) or macrophage inflammatory protein-2-beta (MIP2b)] (also known as IL-1Ra; see **Supplementary Figure 3**) (**Figures 1A–C**). Since aquaporins (AQPs) have been linked to neutrophil deformability during priming and de-priming (24), regulating changes in cell shape and volume, as well as membrane dynamics, neutrophil chemotaxis (25), phagocytosis, and degranulation, we also examined their expression after CBD exposure. We observed only modest variation in the expression levels of both AQP6 and AQP9 across the conditions tested (**Supplementary Figure 3C**). Indeed, analysis of secreted cytokines in the cell supernatants from these ex vivo assays showed nearly complete suppression of TNF-α (**Figure 1D**) and a significant increase in IL-8 levels (**Figure 1E**), with no change in CXCL2 levels (**Figure 1F**). Notably, the cytokine levels of secreted IL-1β or IL-6 were undetectable 2 hours after the cells were exposed to CBD and activated with LPS.

To test whether any of the CBRs (e.g., CBR1 and CBR2) play a role in neutrophil activation and to link the effect of CBD on human neutrophils to a particular CBR, we tested the levels of secreted cytokines following CBD exposure in the presence of AM251 (a CBR1 inverse agonist), SR144528 (a CBR2 inverse agonist) or BCTC (a TRP channel inhibitor) following LPS activation. The CBR2 inverse agonist SR144528 abolished CBD suppression of TNF-α secretion, restoring its overall levels (**Figure 1G**). Unexpectedly, blocking CBR2 signaling strongly increased the IL-8 concentration (**Figure 1H**) but did not affect the CXCL2 concentration (**Figure 1I**).

To further investigate the signaling pathway responsible for CBD’s cellular effects, we used a human MAPK phosphorylation antibody array and subjected protein extracts taken from neutrophils stimulated with LPS (as a baseline activation control) or LPS + CBD (**Figure 1J**). Although many critical cell regulatory signaling pathways commonly studied with validated antibodies are represented in this array, the only noticeable pathway showing a significant change in response to CBD is the serine/threonine protein kinase glycogen synthase kinase 3 alpha (GSK-3α) (**Figure 1J**). Since GSK-3α is constitutively expressed as the predominant isoform in human neutrophils, whereas cell stimulation with fMLP, LPS, and GM-CSF results in the phosphorylation of Ser21, which deactivates its activity (significantly decreases active site availability) (26, 27), we validated the elevated levels of phospho-GSK-3α (Ser21) in CBD-treated neutrophils (e.g., maintain its activity) using different specific antibodies and FACS analysis (**Figures 1K, L**, **Supplementary Figure 4**). Interestingly, TNF-α expression in human neutrophils has already been reported to be governed by GSK-3α, and its inhibition by lithium chloride markedly amplifies TNF-α synthesis and release by human neutrophils (27).

**Figure 2:**
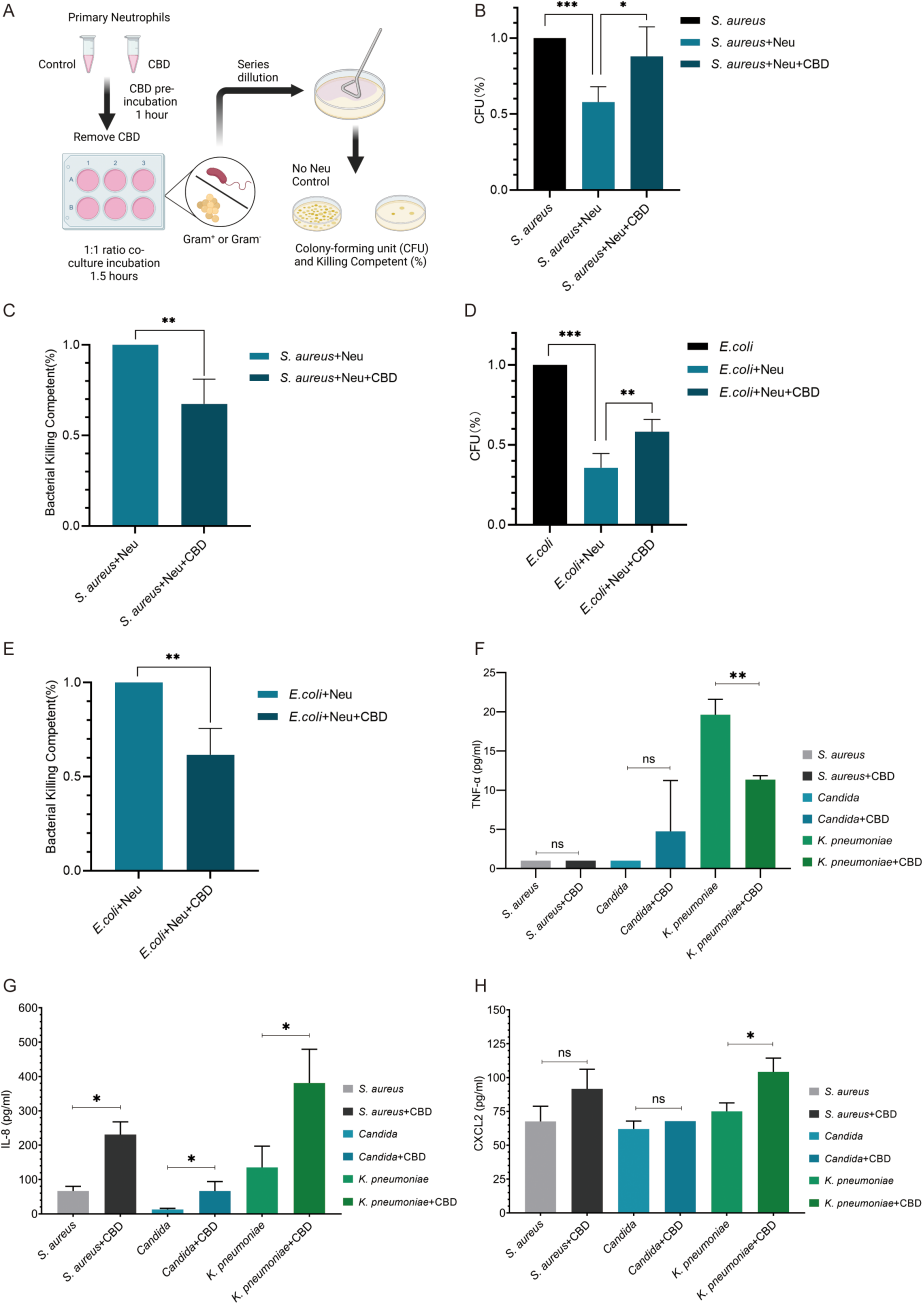
CBD attenuates oxidative burst ROS production in LPS or PMA-activated human neutrophils but does not affect neutrophil extracellular traps. Representative FACS histogram plots of intracellular ROS-based detection of **(A)** LPS-activated or **(B)** PMA-activated neutrophils assessed with the fluorogenic cell-permeant reagent DHR 123. FACS quantification (MFI -/+ SD, n=3) comparing intracellular ROS production in resting (untreated) or **(C)** (25 µg/ml LPS) and **(D)** PMA (100 nM)-activated human neutrophils preincubated with or without CBD (12.5 µM) or with pretreated cells with GPR55 antagonist (CID-16020046) with CBD (12.5 µM). **(E)** Fluorescence microscopy snapshots showing the extent of NETosis achieved by human neutrophils preincubated with or without CBD (12.5 µM) and then activated with either LPS or PMA (as in C, D). Extracellular DNA NETs were visualized with the non-permeable nucleic acid stain green-fluorescent SYTOX Green. **(F)** SYTOX Green-based quantitative plate-based NETosis assay of *ex vivo* NETs (as in E) showing no significant effect of CBD on NET formation. SYTOX Green plate-based *ex vivo* assay for quantifying NETosis induction by **(G)** the potent and highly selective CBR1 agonist ACEA (1 mM) or **(H)** different concentrations of the CBD derivative and CBR2 agonist. * Significant p < 0.05; ** More significant p < 0.01; *** Highly significant p < 0.001; ns, Not significant p ≥ 0.05.

CBD is perceived as an antioxidant that can modulate oxidative signaling (25), so it has been shown to moderately reduce reactive oxygen species (ROS) production in human polymorphonuclear leukocytes (PMNL) (28). We then tested its effect on LPS and phorbol-12-myristate-13-acetate (PMA)-induced neutrophil oxidative bursts. Assessing neutrophil superoxide production in cells pre-exposed to CBD and then activated with LPS (**Figures 2A, C**) or PMA (**Figures 2B, D**) using dihydrorhodamine 123 (DHR 123) assay showed that CBD significantly alleviated LPS or PMA-induced oxidative burst. Since the activation of orphan G protein-coupled receptor 55 (GPCR55) has been linked to the inhibition of oxidative burst in neutrophils and a reduction in ROS production triggered by C5a or CRB2 activation (29), we also examined the possibility that GPR55 may be involved in the observed ROS reduction following CBD exposure, even though CBD is known to block its activation. As expected, the selective GPR55 antagonist CID 16020046 did not influence the reduced ROS production by CBD in LPS or PMA-activated cells (**Figures 2C, D**). Furthermore, released extracellular ROS levels showed a similar pattern of results to those obtained with the DHR123 assay (**Supplementary Figure 5**).

Since ROS production is a central and critical phase required for generating neutrophil extracellular traps (NETosis), we next tested the ability of CBD to impact NET capacity by assessing and quantifying NET production via microscopic (**Figure 2E**) or fluorescence measurement of extracellular DNA by NETosis plate assays (**Figure 2F**). In contrast to recent findings (30), preincubation of neutrophils with CBD did not affect LPS- or PMA-induced NETosis. Moreover, although CBR1 signaling (but not that of CBR2) was reported to induce NETs that mediate MPO release and ROS production in mouse neutrophils, the use of the CBR1-specific agonist ACEA (**Figure 2G**) or the CBR2 agonist Hu-308 (**Figure 2H**) had no significant effect on the formation of purified primary human neutrophil NETs.

**Figure 3:**
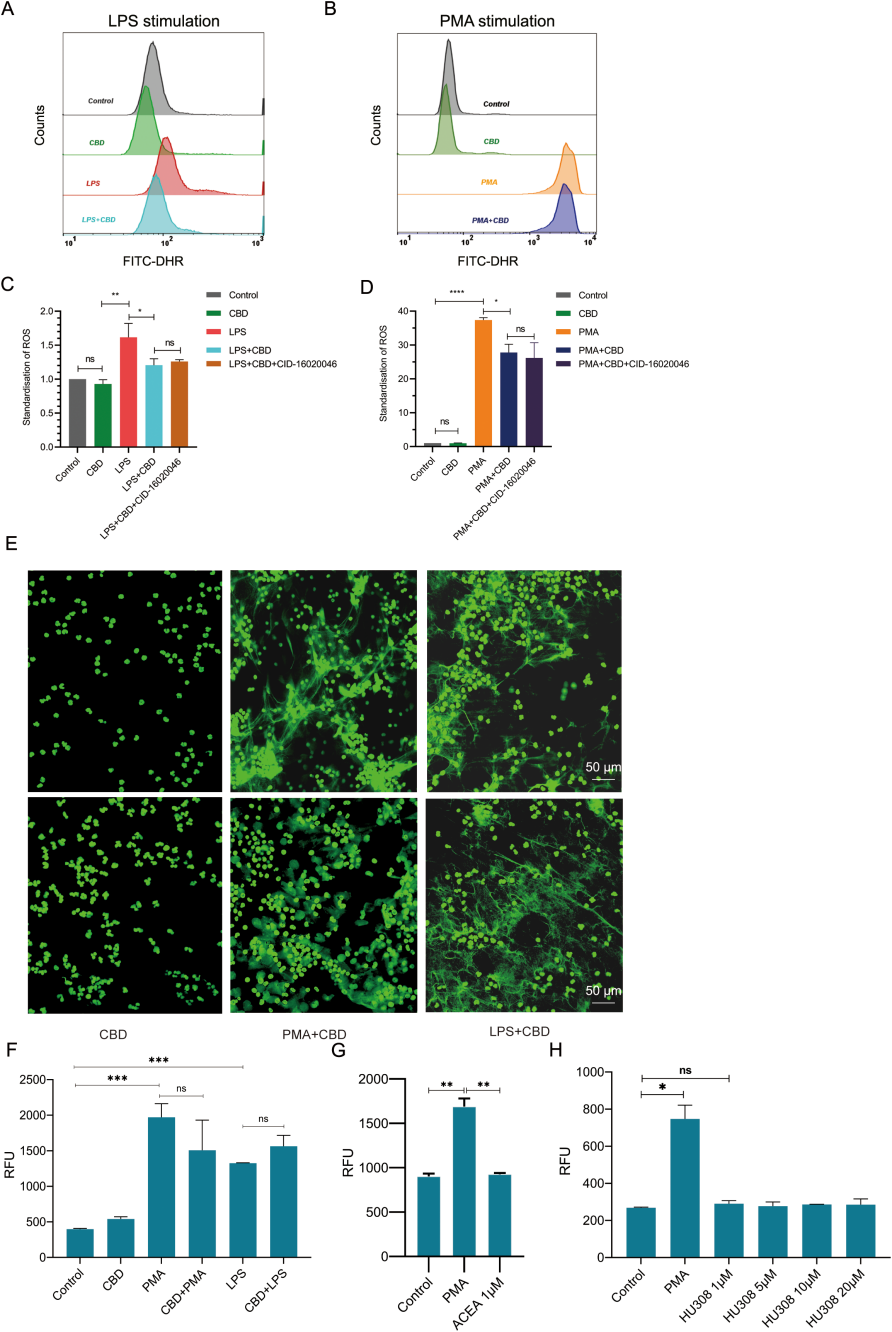
CBD impairs bacterial killing and alters cytokine and chemokine secretion by human neutrophils in response to clinically relevant pathogens. **(A)** Schematic representation of the time-dependent neutrophil-bacteria coincubation killing assay. Bacterial cultures were incubated with neutrophils preincubated with or without CBD at a 1:1 ratio for 1 h. The rates of bacterial clearance by neutrophils and the kinetics of surviving bacteria were monitored to determine the effects of CBD and CBR2 signaling on neutrophil-killing efficiency, overall bactericidal activity, and bacterial survival. **(B)** Bacterial survival and **(C)** neutrophil-killing efficiency of gram-positive *S. aureus* or **(D)** bacterial survival and **(E)** neutrophil-killing efficiency of gram-negative *E*. *coli.* Untreated (resting) or CBD-treated human neutrophils were exposed to heat-killed clinically relevant pathogens such as *S. aureus*, Candida, and *K*. *pneumoniae* strains, and the levels of secreted cytokines in cell supernatants were measured for **(F)** TNF-α **(G)** IL-8 and **(H)** CXCL2. * Significant p < 0.05; ** More significant p < 0.01; *** Highly significant p < 0.001; **** Very highly significant (less common) p < 0.0001; ns, Not significant p ≥ 0.05.

To additionally test whether CBD can also affect neutrophil bacterial killing capacity, we tested the total killing of gram-positive (*S. aureus*) and gram-negative (*E. coli*) bacteria by neutrophils with or without preexposure to CBD. Neutrophils were preincubated with or without CBD for 30 minutes and then washed twice with fresh cell growth media without antibiotics to remove traces of CBD (to avoid its bactericidal effect). Neutrophils and bacteria were mixed at a 1:1 ratio for 1 h to allow bacterial clearance. The remaining uningested extracellular bacteria were separated from the neutrophils by low-speed centrifugation. The killing or bacterial survival percentage rates were determined based on viable counts of colony-forming units (CFU) compared to those of bacterial samples containing no neutrophils as a control (**Figure 3A**). Neutrophils exposed to CBD showed a 3.5-fold (80%) decrease in the ability to kill *S. aureus* (e.g., Neu 42.05% vs. Neu + CBD 12.01%) (**Figures 3B, C**) and an approximately 35% reduction in the killing capacity for *E. coli* (e.g., Neu 64.35% vs. Neu+CBD 41.88%) (**Figures 3D, E**). Consequently, we examined the ability of neutrophils to secrete different cytokines in response to clinically relevant pathogens, such as *S. aureus*, *Candida albicans* (Candida), and *Klebsiella pneumoniae* (*K. pneumoniae*). In line with our previous results, we observed a significant reduction in TNF-α levels (**Figure 3F**), a sharp increase in secreted IL-8 (**Figure 3G**), and a significant increase in CXCL2 (**Figure 3H**) when neutrophils were exposed to heat-killed pathogens in the presence of CBD.

**Figure 4:**
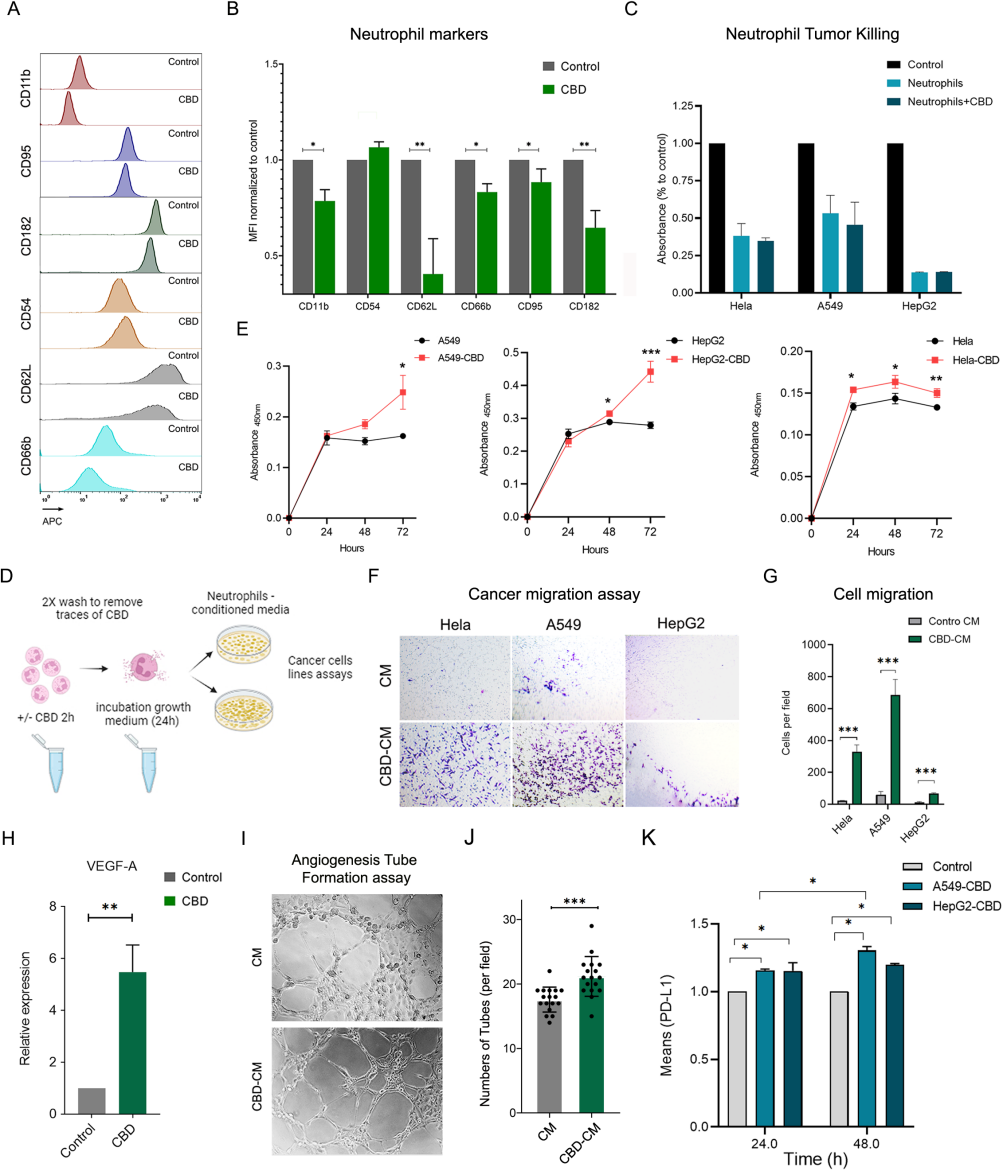
CBD-exposed human neutrophils acquire N-2-like phenotypic polarization features. **(A)** Representative FACS histograms and **(B)** quantification of chosen cell surface expression markers and typical N1 and N2 markers before and after primary human neutrophils were incubated with CBD (12.5 µM for 1 h*) in vitro*. The typical N1 marker FasR (CD95), the intercellular adhesion molecule (ICAM)-1 (CD54), the typical N2 marker CD182 (CXCR2) and the neutrophil activation markers L-selectin (CD62L), CEACAM8 (CD66b) and integrin alpha M (CD11b) were all assessed by FACS analysis and are expressed as the mean fluorescence intensity (MFI)-/+ standard (n=4). **(C)** Neutrophil antitumor effects on A549, HepG2, and HeLa tumor cells were examined via a neutrophil *in vitro* cancer cell-killing assay. Highly pure human neutrophils from healthy donors were preincubated with or without CBD, washed extensively, and cocultured with the different cancer cell lines at a ratio of 20:1 for 24 hours. Following overnight incubation, the signal from the remaining viable cells was measured by an XTT assay and compared to that from non-neutrophil control cancer cell cultures. The data are presented as the means -/+ SDs. of (n=3) different donors. **(D)** Schematic representation of CBD-free or CBD-stimulated human neutrophil conditioned medium. Human neutrophils were preincubated with 12.5 µM CBD for 1 h, washed extensively with 2X to remove traces of CBD, and incubated in fresh neutrophil growth medium for 24–48 h to secrete and release multiple cytokines and chemokines. Untreated cells subjected to the same procedure served as controls. **(E)** Parallel cultures of the human cancer cell lines A549, HepG2, and HeLa were all grown in parallel (96 wells) with neutrophil condition medium, stimulated or unstimulated with CBD, and cell proliferation was monitored every 24 h using an XTT assay. **(F)** Representative light microscope images from the Transwell migration assay showing HeLa, A549, and HepG2 cells incubated overnight with neutrophil CM or CBD-CM, followed by crystal violet staining. **(G)** Quantification of migrated cells per field from the assay in **(F)**. Data represent the mean -/+ SDs of (n=3) different donors. **(H)** Relative mRNA expression level of VEGF-A of neutrophils incubated for 2 h with CBD, normalized to control-untreated cells. **(I–K)** Angiogenesis tube-formation assay. **(I)** Representative light microscopy images of HUVECs in Matrigel ™ assay incubated with neutrophil CM or CM-CBD for 4 h according to the instructions of the Angiogenesis Assay Kit (Abcam, ab204726). **(J)** Mean number of tubes in 5 fields per well for HUVEC treated with CM or CM-CBD of n=3 different donors. **(K)** Treatments with CBD-stimulated neutrophil-conditioned medium elevated the expression of PD-L1 in cancer cells. 1X10^6^ A549 and HepG2 cancer cell lines were grown in neutrophil CM or CM-CBD for 24 and 48 hours. The levels of surface PD-L1 were measured using specific antibody FACS staining and are expressed as the relative (to control) MFI -/+ SD. of (n=3) different donors. * Significant p < 0.05; ** More significant p < 0.01; *** Highly significant p < 0.001.

N2-polarized neutrophils represent a distinct subset with unique characteristics within the spectrum of neutrophil polarization. N2-polarization primarily refers to the neutrophil differentiation state and is often associated with anti-inflammatory and tissue-repairing functions. Since the characteristics of primary human neutrophils exposed to CBD are highly similar to those of N2-type neutrophils (31), as was the increase in secretion of IL-8 and subclasses of chemokines (CCLs and CXCLs); production of low levels of interferon alpha/beta (IFNα/β) and particularly TNF-α ; and decreased ROS production and microbial killing capacity (31), we tested the possibility that CBD and CBR-2 signaling may drive neutrophil polarization toward an N2-like phenotype. To evaluate whether neutrophils exposed to CBD acquire N2-phenotypic features, we examined the levels of several cell surface markers (recently characterized and defined in a novel *in-vitro* polarization protocol) typically expressed by N2-polarized cells. Following the newly developed N2 *in-vitro* polarization protocol, using flow cytometry, we detected a rapid reduction in the expression of activation markers such as L-selectin (CD62L), integrin alpha M (CD11b), CD182 (C-X-C motif chemokine receptor 2 (CXCR2)), CEACAM8 (CD66b), and the expression of the typical N1 marker FasR (CD95), which is also known to regulate neutrophil lifespan (via Fas-mediated neutrophil apoptosis), also significantly decreased following CBD exposure (**Figures 4A, B**). After a short incubation and CBD exposure time (12.5 μM for 150 min), striking compatibility was observed between the tested phenotypic N2-cell surface marker array and the control (**Figures 4A, B**). To further test whether CBD-exposed N2-like polarized cells also share characteristics with *in vivo* N2-polarized neutrophils, we assessed the physiological features of tumor-associated neutrophils (TANs) and whether N2-polarized cells, such as neutrophils, induce antitumor (tumor killing) and tumor-promoting abilities (those that boost tumor proliferation, migration, and angiogenesis) (32, 33). Although CBD did not affect neutrophil cytotoxicity or cancer-killing ability (**Figure 4C**), CBD-free conditioned medium (CM) from neutrophils stimulated with CBD (**Figure 4D**) significantly enhanced cancer cell proliferation (**Figure 4E**) and migration (**Figures 4F, G**). Neutrophils exposed to CBD also showed a four- to fivefold increase in vascular endothelial growth factor A (VEGF-A) expression (**Figure 4H**), and CBD-conditioned medium (CBD-CM) strongly induced tube formation in human umbilical vein endothelial cells (HUVECs) in an *in-vitro* angiogenesis tube formation assay (**Figures 4I, J**). Finally, CBD-CM also elevated (20—25%) the expression of the pro-tumorigenic immune checkpoint inhibitor PD-L1 in cancer cells (**Figure 4K**), suggesting that this signaling pathway may also be involved in neutrophil-mediated suppression of T-cell immunity (34, 35).

**Figure 5:**
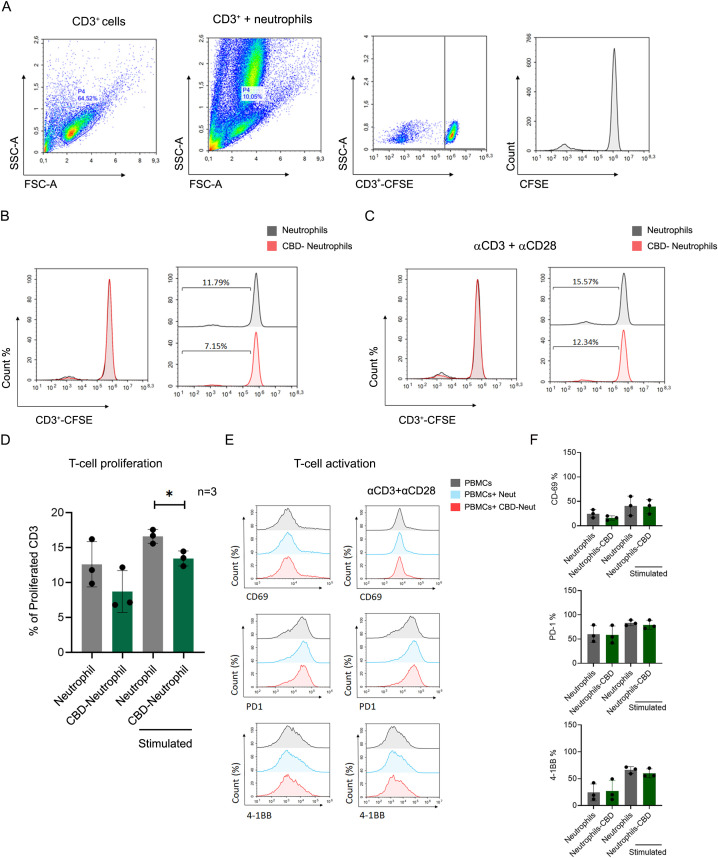
CBD-stimulated neutrophils suppress T-cell proliferation. CFSE-labeled healthy donor PBMCs (CD3^+^) were costimulated and incubated with human primary neutrophils +/- CBD. Coculture with CBD-stimulated neutrophils impaired proliferation. **(A)** The gating strategy of the coculture experiment. Representative individual FACS plot showing CFSE-labeled CD3^+^ cells induced by monoclonal anti-CD3 and anti-CD28 antibodies, for which **(B)** unstimulated or **(C)** T-cell expansion was activated via the αβ-T-cell receptor. **(D)** Quantification of the percentage of cell proliferation (n=3). The data are expressed as the means +/- SDs of 3 independent donors. **(E)** Neither direct cell-to-cell contact nor +/- CBD neutrophil conditioned media (CM) altered the expression of T-cell activation markers (CD69, PD-1, or 4-1BB). **(F)** Quantification of the expression levels of T-cell activation markers (CD69, PD-1, and 4-1BB). Data are presented as mean ± SD from three independent donors.

Indeed, while neutrophils are traditionally known for their role in the innate immune response, recent findings highlight their significant influence on adaptive immunity, mainly through interactions with T-cells (31, 32). Neutrophils have been found to suppress T-cell functions by inducing apoptosis or impairing proliferation (31, 33–35), and activation (31, 35). Given their prevalence in solid tumors and varying phenotypes in different tumor environments, we explored whether CBD-stimulated N2-polarized neutrophils might also impact T-cell functions or hinder antitumor T-cell immunity. We assessed the proliferation of human peripheral CD3^+^ T-cells labeled with carboxyfluorescein succinimidyl ester (CFSE) (**Figure 5A**), cocultured with neutrophils with or without CBD pre-exposure in the absence (**Figure 5B**) or presence of T-cell proliferation stimulation with anti-human monoclonal CD3 and CD28 antibodies (**Figure 5C**). T-cell proliferation was significantly lower (approximately 25-30%) in the presence of CBD-stimulated cells than in the absence of CBD-stimulated neutrophils (**Figure 5D**). Surprisingly, the expression of neutrophil suppressive factors such as arginase-1 (Arg-1), matrix metallopeptidase 9 (MMP-9) and programmed death-ligand 1 (PD-L1) (CD274) was unchanged or even decreased (**Supplementary Figure 6**), and neutrophil/T-cell coculture seemed not to affect the expression of T-cell activation markers either directly (through cell-to-cell contact) or indirectly (through secreted factors after neutrophil CBD-CM) (**Figures 5E, F**).

In conclusion, our study provides compelling evidence and elucidates yet unknown neutrophil polarization signaling pathways involving CBD. These molecular mechanisms underlying neutrophil polarization shed new, unexpected light on the role of the ECS and cannabinoids in neutrophil biology and, on the one hand, hold immense clinical importance or, on the other hand, significant therapeutic potential, opening new avenues for targeted interventions and modulation of neutrophil behavior in inflammatory disorders, autoimmune diseases, and cancer immunotherapy.

## Discussion

CBD functions as a multitarget pharmaceutical agent that primarily acts as an inverse agonist of CBR1 in the central nervous system and CBR2 in immune cells. (4, 36). Due to its molecular flexibility, CBD can interact with multiple other metabotropic receptors, including the GPR55 (4); the serotonin receptors 5-HT1A and 5-HT2A; adenosine receptors such as A1 and A2; and the TRP cation channel subfamily (which includes TRPV1–4 and TRPA1 channels with antagonistic effects on the TRPM8 channel) (37, 38). While consistently shown to be anti-inflammatory, most CBD studies have revealed that CBD’s immunomodulatory effects (including reducing neutrophil migration) primarily involve mouse models and therefore cannot be directly translated to human cells. For example, both mouse and human neutrophils have been shown to express CBR1 and CBR2; however, it is essential to recognize that the different species have fundamental variations in their expression (39, 40) and, therefore, findings from animal studies should be carefully evaluated in the context of animal physiology and may not be directly translated to humans (41, 42). At the same time, CBD has indeed been shown to inhibit cytokine production (in both animal and human cells) during innate and adaptive immune responses, and CBR1/2 activation is primarily associated with neutrophil chemotaxis and migration inhibition, as well as a reduction in their proinflammatory responses. (4, 14, 39, 40, 43). Overall, these observations strongly support the notion that components and signals generated by the ECS can regulate neutrophil activity. Nonetheless, the specific role of the ECS in neutrophil physiology, particularly in CBR-1/2 signaling, remains undetermined mainly, and the detailed mechanisms or long-term consequences of CBD’s influence on the human immune system and cells require further elucidation. Here, we comprehensively assessed the effect of CBD on highly pure primary human neutrophils and showed its effect and overall influence on a range of human neutrophil functions and its collateral impact on tumor cells.

In agreement with a recent report showing that CBD induces cytoplasmic vacuolization, proapoptotic nuclear condensation, and a significant decrease in the viability of human polymorphonuclear cells (22), we also demonstrate that CBD induces extensive neutrophil apoptosis (*ex-vivo*) in a time- and dose-dependent manner (above 15 μM) (**Supplementary Figure 1**). Therefore, it is important to consider the context and effective CBD dosage for each cellular function to understand the full physiological impact of CBD on a particular cell. Accordingly, in line with prior observations investigating the pharmacological and biological impacts of CBD on immunomodulation and angiogenesis, as well as the modulation of neuronal and cardiovascular functions (44), our study also demonstrated that CBD exerts its effects on human neutrophils through CBR-2-dependent and CBR-2-independent mechanisms. For instance, the proapoptotic properties of CBD are CBR-1/2 independent (**Supplementary Figure 1**), while the alterations in cytokine production and secretion (TNFα) (**Figure 1**) seem to be sensitive to CBR-2 blocking. On the other hand, contrary to the preceding mechanism, suggesting that CBD acts only on activated cells without any effects on physiological cell homeostasis (resting cells) (45), our results clearly show that unstimulated neutrophils also respond to CBD and CBR-2 blocking. At the molecular level, our cytokine transcription panels (also reflected by the ELISA) showed substantial changes in cytokine transcript levels in nonactivated cells exposed to CBD, in which IL-8, CCL1-4, and CXCL1–3 were induced, and TNF-α and IFBα/β were repressed (**Figure 1**).

The ability of CBD to modulate both pro- and anti-inflammatory cytokine production and secretion has been widely reported, mainly in monocytes and macrophages (4). It is subsequently suggested that CBD might exert an immunoregulatory influence, helping to equilibrate immune activation and leading to a more balanced immune response (10). Notably, despite typical variability in human primary cell studies, we surprisingly found minimal inter-donor differences in CBD responsiveness. The consistent trends in CBD-induced neutrophil polarization and functional modulation suggest a broad, generalizable effect, most likely driven by CBD’s receptor-independent mechanisms, and therefore, not affected by variable CBRs expression in different individuals. While a detailed analysis of individual donor variations could yield additional valuable information, our current findings establish a robust baseline for future investigations into these nuances and support the possibility of future clinical use of CBD as an immunomodulatory agent. CBD’s ability to interact with multiple immune system cells, most probably at the molecular level, also highlights its potential therapeutic applications in conditions associated with dysregulated immune responses. Although human clinical validation would greatly strengthen our *ex-vivo* findings of CBD-induced human neutrophil polarization, substantial ethical concerns complicate its pursuit, stemming from growing reports, including our research and that of others, indicating potential adverse effects of CBD or cannabis use, especially in cancer or immunocompromised patients. However, the *in vivo* observation of N2-neutrophil polarization by CBD in a mouse model strongly supports our findings, despite the inherent physiological differences between species (46).

At the cellular level, CBD signaling dramatically altered the landscape of surface receptor expression, as indicated by the observed N2 polarization characteristics (**Figures 4A, B**). While CBD is known to inhibit neutrophil ROS generation *in vivo* in animal models (47), our *ex vivo* results using primary human neutrophils also showed that LPS or PMA-activated neutrophils pre-exposed to CBD exhibited a significant reduction in the capacity for ROS generation (**Figure 2**, **Supplementary Figure 5**). Contrary to our expectations, the reduction in ROS production was surprisingly inadequate to inhibit or affect NET formation induced by PMA or LPS (**Figure 2**). However, it likely contributed to the reduced bacterial killing of gram-positive and gram-negative bacteria by CBD-treated neutrophils (**Figure 3**). This can most likely be explained by the artificial *ex-vivo* neutrophil activation procedure, which utilizes high levels of PMS (100 nM) or LPS (25 μg) to trigger NETosis, visualized by fluorescence microscopy.

Like many other vital cells in the immune system, neutrophils also display distinct polarization states. The N2 neutrophil polarization state is a notable phenotype associated with the anti-inflammatory response and tissue repair. Indeed, characterized by their anti-inflammatory profile in many autoimmune diseases and injuries, N2-polarized neutrophils help attenuate excessive immune responses, providing a regulatory mechanism that prevents collateral damage and contributes to inflammation resolution and participation in wound healing. On the other hand, in the context of cancer, N2-polarized cells seem to play a significant role in the tumor microenvironment (TME) when they are present as TANs in the TME milieu and are associated with tumor-promoting effects. As such, N2-polarized neutrophils can facilitate and promote tumor proliferation, immunosuppression, angiogenesis, and extracellular matrix remodeling, ultimately fostering an environment conducive to tumor growth and progression (48). Furthermore, considering the pivotal pro-tumorigenic roles of IL-8 (41, 42), and other factors found in CBD-stimulated neutrophil CM, like VEGF, and CXCL2 (44) in promoting cancer progression [reviewed in (45)], it is no wonder that the CBD-CM (rich in IL-8, VEGF and several chemokines), boosted cell growth, migration, angiogenesis and elevated the levels of cancer cell PD-L1 (**Figure 4**). Interleukin-8 (IL-8), for instance, exhibits a potent pro-angiogenic activity that facilitates the formation of new blood vessels within tumors, serving as a critical supply line for sustained cancer growth and expansion. This enhanced vascularity also permits tumor cells to enter the bloodstream and metastasize. Furthermore, elevated levels of IL-8, in conjunction with its signaling through CXCR1 and CXCR2, directly promote tumor cell proliferation and survival by accelerating the cell cycle and inhibiting apoptosis (49). In addition to growth, increased IL-8 levels enhance metastasis and invasion by augmenting cell motility, migration, and the epithelial-mesenchymal transition (EMT) (49). Beyond its well-recognized role in neutrophil recruitment and NETosis—both of which contribute to cancer progression—IL-8 orchestrates the recruitment and modulation of immunosuppressive immune cells, such as myeloid-derived suppressor cells (MDSCs) (49). As a consequence, high levels of IL-8 within the TME are associated with resistance to chemotherapy and radiotherapy. Conversely, TNF-α deficiency in the TME can reduce tumor cell death and hinder the recruitment and activation of anti-tumor immune cells, thereby limiting the infiltration of essential effector cells (50). The resulting TME, seemingly shaped by the N2-polarized neutrophils, characterized by elevated IL-8 and diminished TNF-α, presents a formidable combination of pro-angiogenic, pro-metastatic, and immunosuppressive signals, thereby establishing an inhospitable environment for anti-tumor responses and significantly enhancing tumor survival and growth.

In light of our previous clinical findings showing that cannabis consumption may hinder cancer immunotherapy and the strong reports of Delta-9-tetrahydrocannabinol (THC) involved in CBR2 signaling in CD8+ T-cells and NK suppression (51–53), combined with our observation that CBD-stimulated neutrophil/T-cell suppresses T-cell proliferation (**Figure 5**), it is tempting to speculate that combined blockade of the IL-8/IL-8R axis with ICI immunotherapy could ultimately improve antitumor T-cell efficacy (45). Moreover, inhibiting CBR2 may aid in suppressing tumor growth and enhancing anti-tumor immunity across various immune targets, thus preventing the suppression of CD8+ T-cells and NK-cells or the pro-tumorigenic activity of N2-neutrophils. Interestingly, aside from neutrophils, which appear to be polarized through CBR2 signaling, monoacylglycerol lipase (MAGL) deficiency induces M2-like macrophage polarization via endogenous 2-AG-CBR2 signaling in tumor-associated macrophages (TAMs), regulating tumor-associated CD8+ T-cells and fostering cancer progression (54). Furthermore, recent innovative experimental methodologies explore novel strategies to harness and enhance neutrophils’ anti-tumor activity by leveraging neutrophil functional reprogramming. These approaches, such as neutrophil-trained immunity (55) or neutrophil-activating therapy (56), exploit neutrophils’ inherent plasticity, aiming to activate or reprogram neutrophils through targeted interventions, directly boosting neutrophils’ cytotoxic capabilities against cancer cells. It is needless to say that such reprogramming involves critical metabolic and epigenetic alterations, processes that CBD-induced polarization could significantly influence or counteract. Regardless of the approach taken, our findings emphasize the necessity of comprehending the dynamics of CBD-based N2-neutrophil polarization and its intricate balance prior to utilizing its potential for developing treatments aimed at modulating neutrophil behavior.

Consequently, our study lays the groundwork for future investigations into the pharmacological and molecular mechanisms that underpin the effects of cannabis chemical derivatives, particularly emphasizing the influence of CBD on inflammatory disorders. These findings may facilitate further exploration and the development of targeted therapeutics that address CBRs or endocannabinoids for the treatment of various human diseases.

## Data Availability

The original contributions presented in the study are included in the article/**Supplementary Material**. Further inquiries can be directed to the corresponding authors.

## Supplementary material

Supplementary Figure 1 CBD leads to reduced neutrophil viability through the induction of cannabinoid receptor (CBR)-independent apoptotic cell death. (A-D) Time-dependent Annexin V and PI staining and total viability (normalized to untreated control cells) of primarily isolated human neutrophils exposed to increasing CBD concentrations. Isolated pure human neutrophils were exposed to high CBD concentrations in the presence of selective CBR1 (AM-251) or CBR2 (SR144528), or GPR55 (CID-16020046) blockers. (E) Apoptosis or (F) viability levels are indicated as the means -/+ SDs (n=3).

Supplementary Figure 2 CBD does not alter neutrophil morphology. Neutrophils were treated with or without CBD at 12.5 µM for 1.5 or 3 hours, followed by Giemsa stain.

Supplementary Figure 3 CBD alters the gene expression of resting or LPS-activated human neutrophils. The expression levels of various pro- or anti-inflammatory factors, including **(A, B)** cytokines and chemokines, as well as key functional neutrophil effector proteins such as **(C)** aquaporin, were assessed via quantitative PCR (qPCR). The data are presented as the means ± SD (n = 5).

Supplementary Figure 4 Total unphosphorylated GSK-3 protein levels do not change in response to CBD treatment. A representative FACS histogram showing the mean fluorescence intensity (MFI) of a control intracellular stain using an unphosphorylated GSK-3 antibody, showing equal global GSK-3 levels in human neutrophils with or without CBD treatment. The histogram values correspond to the MFI ± SD of n=3 donors.

Supplementary Figure 5 CBD also attenuates extracellular ROS production in LPS or PMA-activated neutrophils. Measurement of absorbance at 570 nm for extracellular H_2_O_2_ using the Amplex Red substrate in the presence of horseradish peroxidase (HRP). **(A)** Cells were treated with CBD alone, LPS alone, LPS with CBD, or LPS with CBD in the presence of the GPR55 antagonist CID-16020046. **(B)** Cells were treated with CBD alone, PMA alone, PMA with CBD, or PMA with CBD in the presence of the GPR55 antagonist CID-16020046. Data are presented as the means +/- SDs or n=3 different donors.

Supplementary Figure 6 Quantification of neutrophil factors after CBD exposure. Highly purified neutrophils were incubated for 2 h with or without CBD 12.5µM **(A, B),** showing relative mRNA expression level of MMP9, Arg-1, and PD-1L. **(C)** A representative FACS histogram showing the mean fluorescence intensity (MFI) of intracellular PD-L1 staining using a specific antibody.

## Glossary

ECS: Endocannabinoid system
CBD: Cannabidiol
CBRs: Cannabinoid receptors
FDA: Food and Drug Administration
EMA: European Medicines Agency
MDSCs: Myeloid-derived suppressor cells
EDTA: Ethylenediaminetetraacetic acid
FBS: Fetal bovine serum
DMEM: Dulbecco’s Modified Eagle Medium
PI: Propidium iodide
FACS: Fluorescence-activated cell sorting, ROS, Reactive oxygen species
DHR123: Dihydroergotamine 123
LPS: Lipopolysaccharide
qPCR: Real-time quantitative polymerase chain reaction
OD: Optical density
CFU: Colony-forming units
RFUs: Relative Fluorescence Units
MFI: Mean fluorescence intensity signals
SD: Standard deviation
NETs or NETosis: Neutrophil extracellular NETs
ELISA: Enzyme-Linked Immunosorbent Assay
CXCL: The chemokine (C-X-C motif) ligand
IL-1: Interleukin-1
PD-L1: Programmed death-ligand 1
TME: Tumor microenvironment
TANs: Tumor-associated neutrophils
GPR55: G protein-coupled receptor 55
MAGL: Monoacylglycerol lipase
TAMs: Tumor-associated macrophages.
