# Supplementary figures and images for: Cannabidiol polarizes human neutrophils toward a cancer-promoting phenotype
Source: Front Immunol. 2025 Jul 25;16:1543403. doi: 10.3389/fimmu.2025.1543403 (PMC12331714; doi:10.3389/fimmu.2025.1543403)

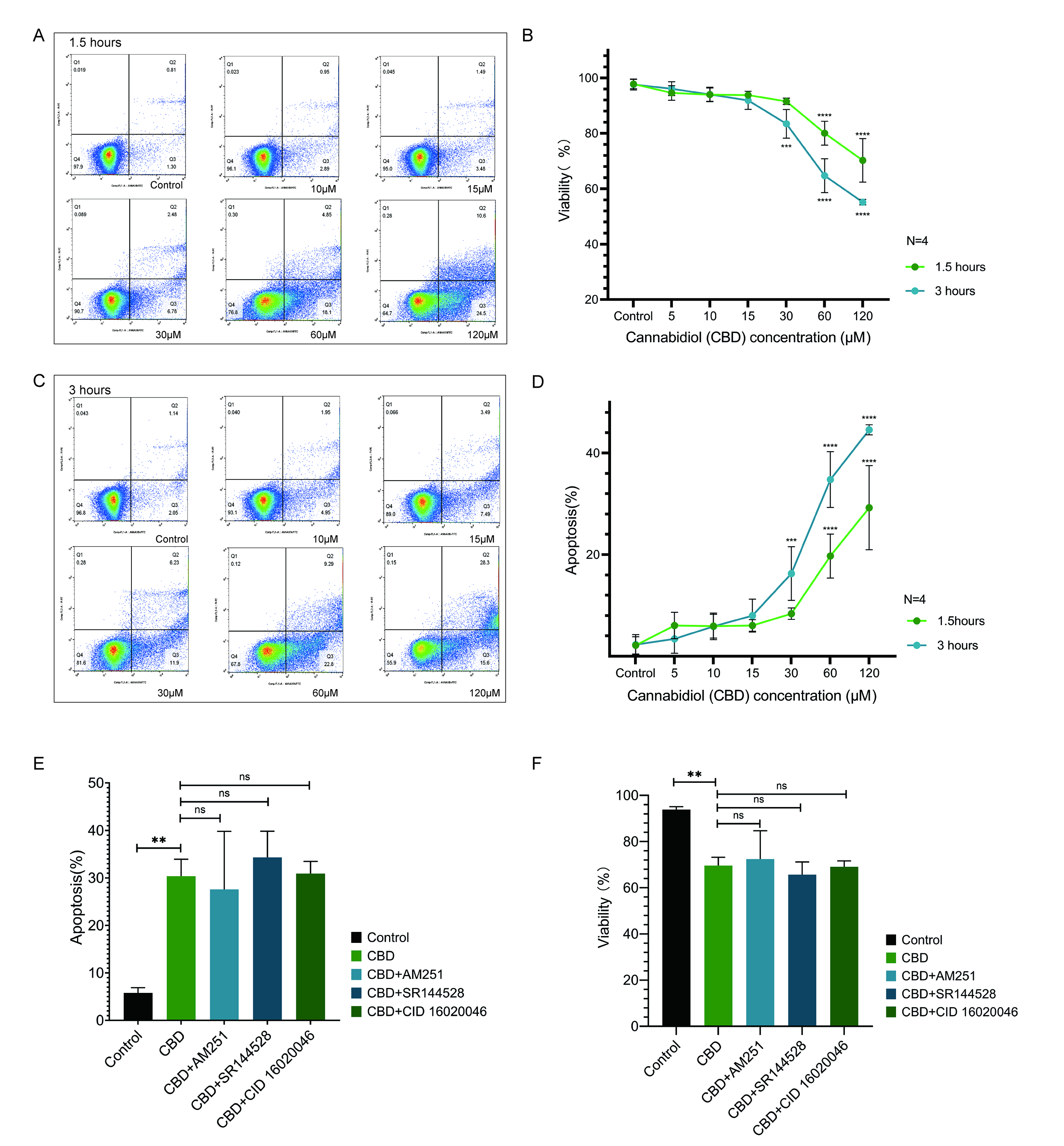

Supplement: Supplementary Figure 1 — CBD leads to reduced neutrophil viability through the induction of cannabinoid receptor (CBR)-independent apoptotic cell death. (A-D) Time-dependent Annexin V and PI staining and total viability (normalized to untreated control cells) of primarily isolated human neutrophils exposed to increasing CBD concentrations. Isolated pure human neutrophils were exposed to high CBD concentrations in the presence of selective CBR1 (AM-251) or CBR2 (SR144528), or GPR55 (CID-16020046) blockers. (E) Apoptosis or (F) viability levels are indicated as the means -/+ SDs (n=3). [file Image1.tif]

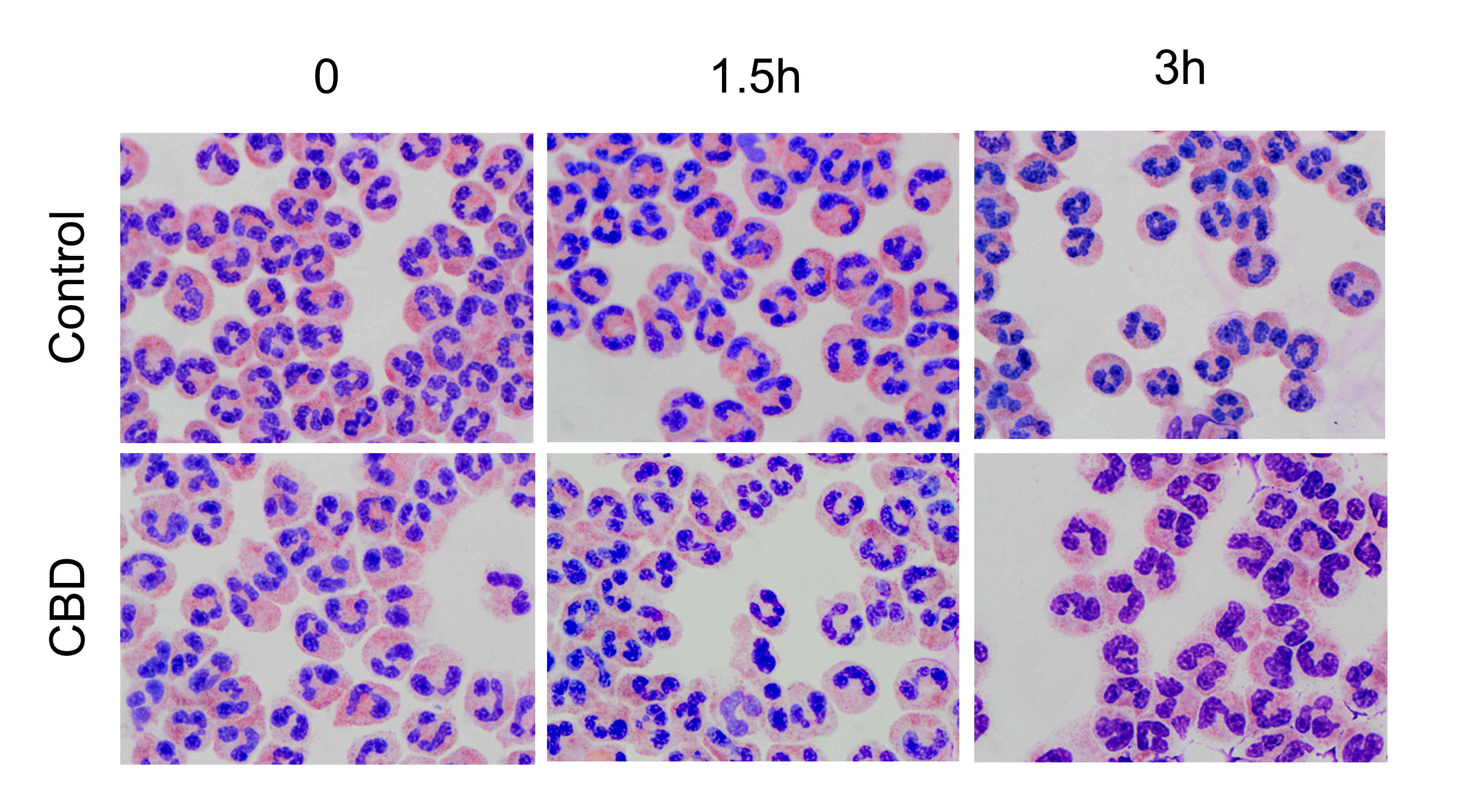

Supplement: Supplementary Figure 2 — CBD does not alter neutrophil morphology. Neutrophils were treated with or without CBD at 12.5 µM for 1.5 or 3 hours, followed by Giemsa stain. [file Image2.tif]

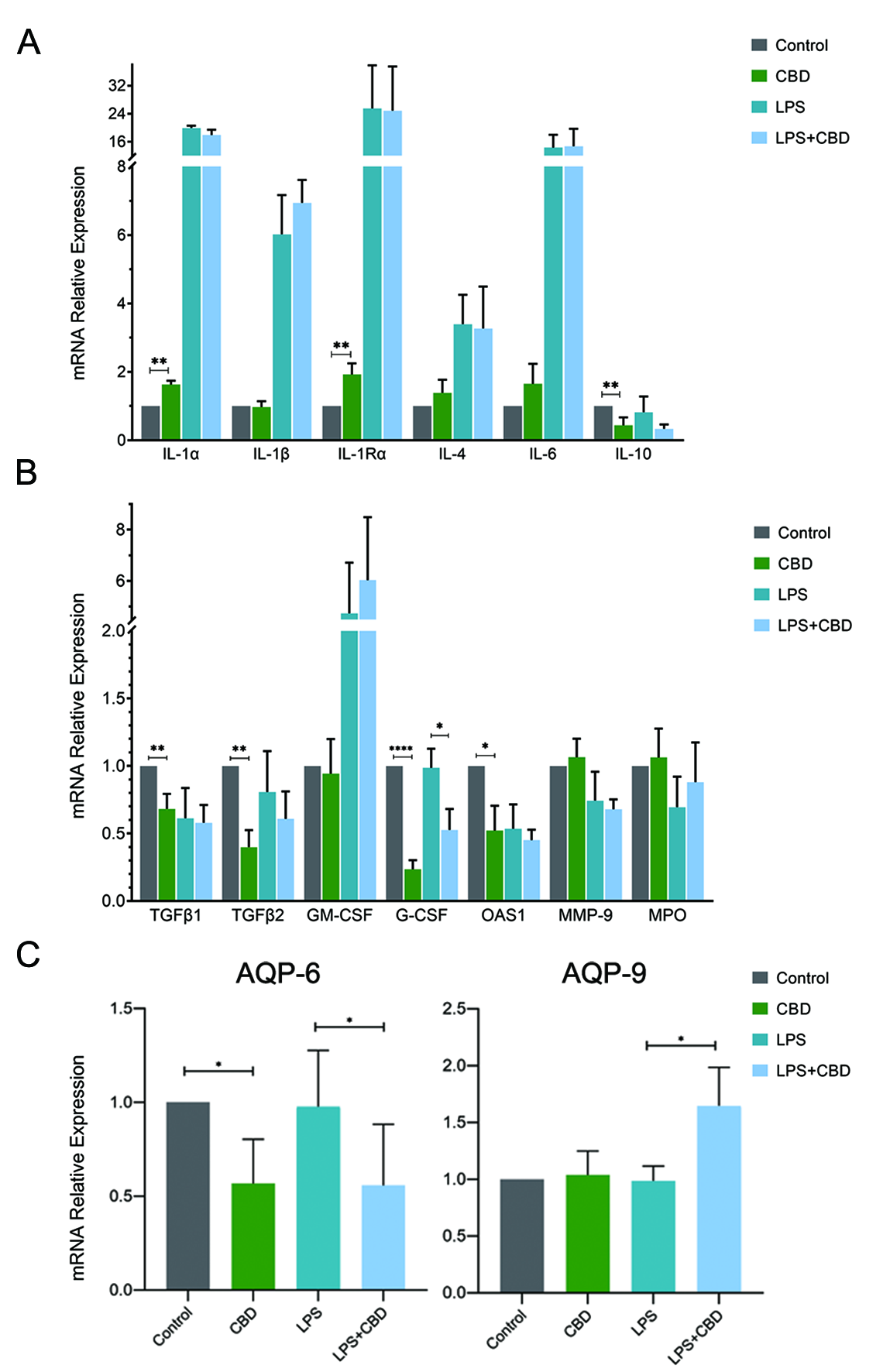

Supplement: Supplementary Figure 3 — CBD alters the gene expression of resting or LPS-activated human neutrophils. The expression levels of various pro- or anti-inflammatory factors, including (A, B) cytokines and chemokines, as well as key functional neutrophil effector proteins such as (C) aquaporin, were assessed via quantitative PCR (qPCR). The data are presented as the means ± SD (n = 5). [file Image3.tif]

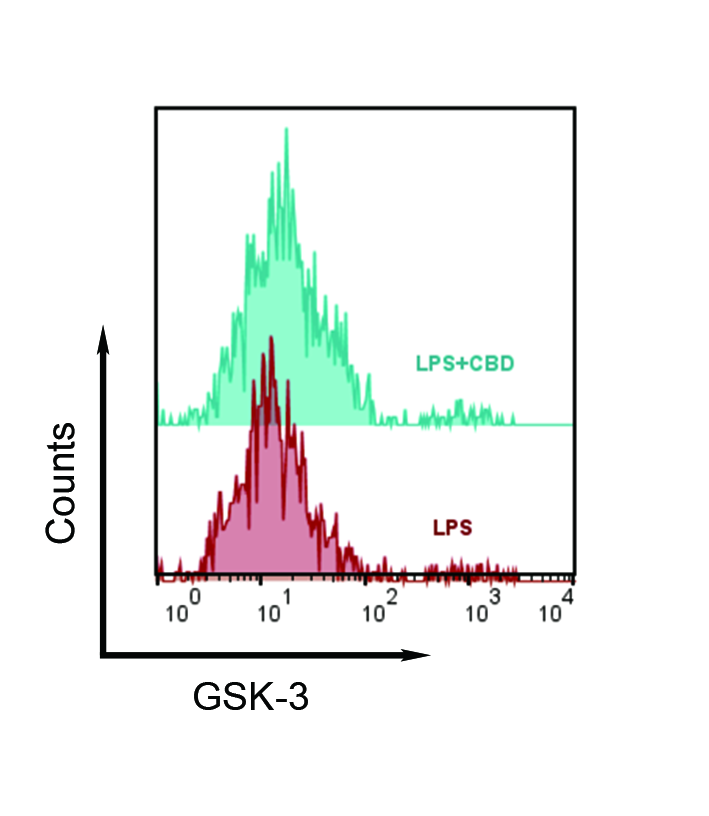

Supplement: Supplementary Figure 4 — Total unphosphorylated GSK-3 protein levels do not change in response to CBD treatment. A representative FACS histogram showing the mean fluorescence intensity (MFI) of a control intracellular stain using an unphosphorylated GSK-3 antibody, showing equal global GSK-3 levels in human neutrophils with or without CBD treatment. The histogram values correspond to the MFI ± SD of n=3 donors. [file Image4.tif]

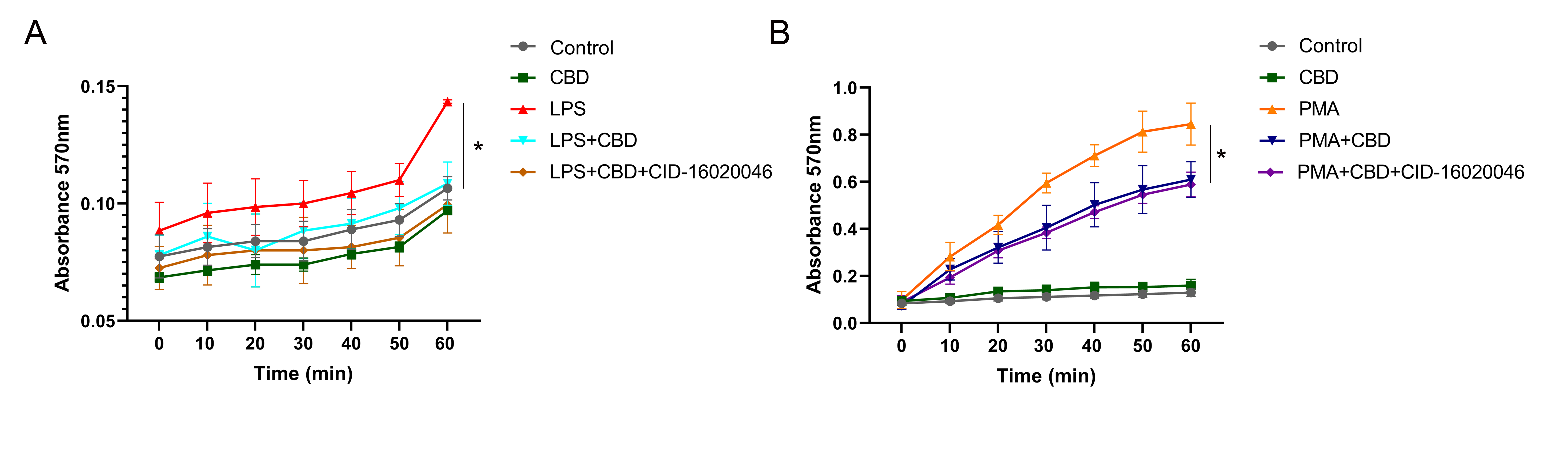

Supplement: Supplementary Figure 5 — CBD also attenuates extracellular ROS production in LPS or PMA-activated neutrophils. Measurement of absorbance at 570 nm for extracellular H2O2 using the Amplex Red substrate in the presence of horseradish peroxidase (HRP). (A) Cells were treated with CBD alone, LPS alone, LPS with CBD, or LPS with CBD in the presence of the GPR55 antagonist CID-16020046. (B) Cells were treated with CBD alone, PMA alone, PMA with CBD, or PMA with CBD in the presence of the GPR55 antagonist CID-16020046. Data are presented as the means +/- SDs or n=3 different donors. [file Image5.tif]

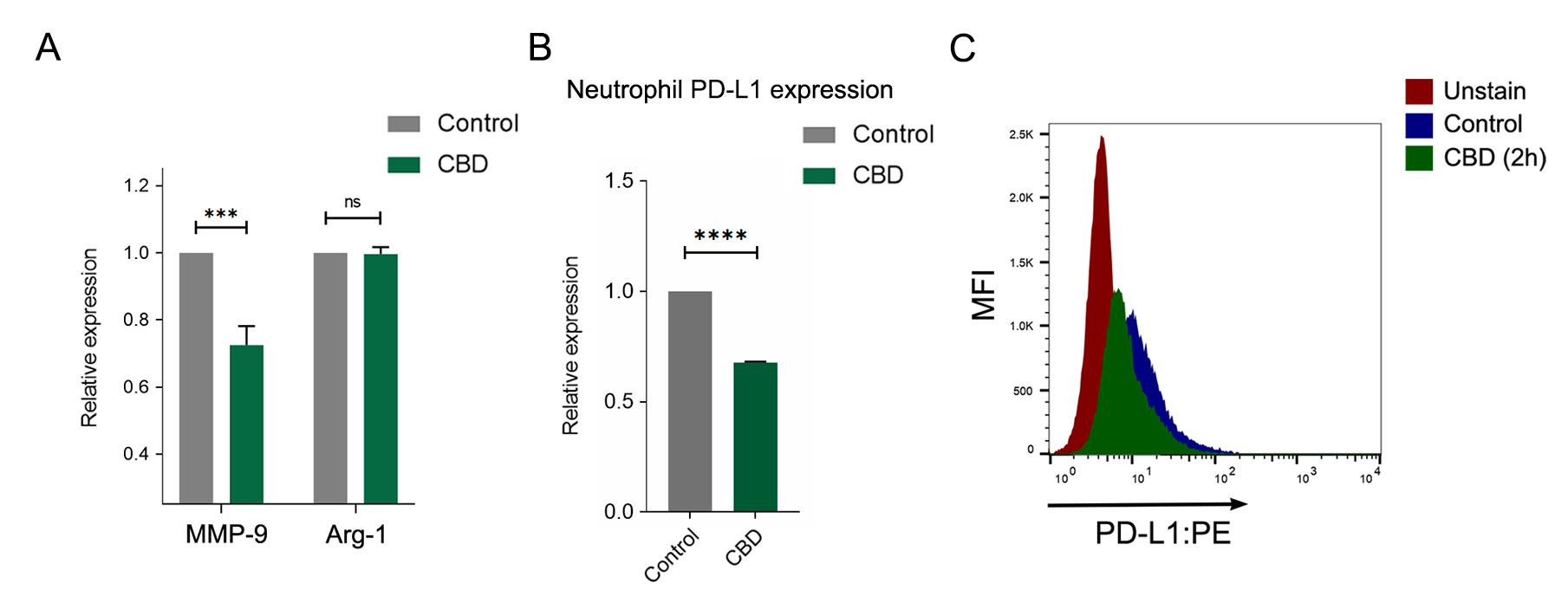

Supplement: Supplementary Figure 6 — Quantification of neutrophil factors after CBD exposure. Highly purified neutrophils were incubated for 2 h with or without CBD 12.5µM (A, B), showing relative mRNA expression level of MMP9, Arg-1, and PD-1L. (C) A representative FACS histogram showing the mean fluorescence intensity (MFI) of intracellular PD-L1 staining using a specific antibody. [file Image6.tif]
